# A Sudden Expansion: Acute Subdural Hematoma From Extramedullary Acute Myeloid Leukemia

**DOI:** 10.7759/cureus.90114

**Published:** 2025-08-14

**Authors:** Soumajit Kundu, Brittany Ewing, Matthew Bower, Madihah Hepburn

**Affiliations:** 1 Department of Medicine, Summa Health, Akron, USA; 2 Department of Pathology, Summa Health, Akron, USA; 3 Department of Neurosciences, Summa Health, Akron, USA

**Keywords:** acute myeloid leukemia, antineoplastic combined chemotherapy protocols, azacitidine, central nervous system neoplasms, extramedullary hematopoiesis, intrathecal chemotherapy, myeloid sarcoma, subdural hematoma, tp53 mutations, venetoclax

## Abstract

Chloromas are extramedullary agglomerations of malignant myeloid blast cells, also known as myeloid sarcoma. Myeloid sarcoma of the central nervous system (CNS) is a very rare presentation of extramedullary leukemic mass infiltration. This may be seen as an intracranial mass due to invasion of the brain parenchyma or as leukemic infiltration of the meninges. Such infiltration may present as a subdural hematoma (SDH), particularly in coagulopathic or immunocompromised patients undergoing chemotherapy.

We present a 63-year-old male with acute myeloid leukemia (AML), previously treated with azacitidine/venetoclax induction and intrathecal methotrexate, who was admitted with abdominal thromboses and started on therapeutic anticoagulation. The patient later developed acute encephalopathy, and imaging demonstrated a large left-sided acute-on-subacute SDH with mass effect. Despite anticoagulation reversal and surgical intervention, the patient experienced recurrent hemorrhage. Histopathology of the subdural membrane tissue confirmed AML with monocytic differentiation. The clinical course was further complicated by tumor lysis syndrome, metabolic acidosis, and acute liver failure. The patient expired following withdrawal of life-sustaining treatment.

This case highlights a rare, catastrophic CNS manifestation of AML in the form of a dural-based chloroma inducing a subacute-chronic SDH. Dural metastasis should be considered in patients with hematologic malignancies and subacute SDH. Although SDHs can typically be treated with good outcomes following surgical evacuation, those due to leukemic infiltration from AML - especially high-risk phenotypes - may portend less favorable outcomes, due to concomitant coagulopathy and being a marker for more aggressive hematologic disease.

## Introduction

Acute myeloid leukemia (AML) is a type of blood cancer characterized by the uncontrolled growth of immature white blood cells, called myeloid precursors. These abnormal cells primarily accumulate in the bone marrow and bloodstream, but may also spread to other parts of the body. Common symptoms at diagnosis include low blood counts (cytopenias), which can cause fatigue, increased infections, and bleeding due to bone marrow failure [1]. AML can also manifest as extramedullary soft tissue masses, known as myeloid sarcoma or chloroma. These tumors of immature myeloid cells can occur at any anatomic site outside of the bone marrow. The most common extramedullary sites are lymph nodes, skin, soft tissue, testes, or gastrointestinal tract. Central nervous system (CNS) involvement is exceptionally rare, reported in only 2%-8% of AML patients [2].

CNS myeloid sarcoma or chloroma is typically contiguous with the meninges or ependyma in the brain or spinal cord, and may present as dural-based intracranial masses, epidural spinal lesions, or, rarely, as intraparenchymal infiltration [3]. Although these patients may present with focal neurologic deficits and seizures due to mass effect, the diagnosis is challenging and often delayed, as CNS chloromas may be radiographically subtle or mimic more common intracranial lesions. Their imaging characteristics frequently resemble those of meningiomas, metastatic tumors, or subdural collections caused by infection or trauma, leading to frequent misdiagnosis. Consequently, histopathologic confirmation is necessary for accurate diagnosis [4-6]. Clinical suspicion is especially low in non-promyelocytic AML subtypes or in patients who have achieved remission following induction chemotherapy [7,8].

A further diagnostic challenge arises when CNS chloromas occur in the setting of chemotherapy-induced cytopenias and coagulopathies, increasing the risk for spontaneous intracranial hemorrhage or extra-axial hemorrhage from minimal trauma. Thrombocytopenia, disseminated intravascular coagulation (DIC), and direct leukemic infiltration of intracranial vasculature can predispose patients to hemorrhagic events, including subdural hematoma (SDH). However, a chloroma causing an SDH is particularly rare, with only isolated cases reporting this phenomenon. In such cases, the clinical presentation may mimic spontaneous or traumatic SDH, especially in patients on anticoagulation or with thrombotic complications [9,10].

The risk of hemorrhagic complications is also higher during the induction phase. The introduction of hypomethylating agents in combination with BCL-2 inhibitors, such as azacitidine and venetoclax, has drastically changed the therapeutic landscape for older patients with AML or those who are poor candidates for intensive chemotherapy [11,12]. While effective, these regimens often result in profound and prolonged cytopenias, placing patients at higher risk of infection, bleeding, and thromboembolic complications during and after treatment [13,14]. Leukemic CNS involvement post-treatment is not well-characterized, especially when presenting with acute neurologic deterioration and radiographic evidence of SDH [15].

## Case presentation

A 63-year-old male was recently diagnosed with AML with monocytic differentiation, confirmed by bone marrow biopsy and flow cytometry. Next-generation sequencing (NGS) revealed mutations in *TP53*, *NPM1*, *PTPN11*, and *RUNX1T1* - features indicative of high-risk AML. He had completed a 28-day cycle of induction chemotherapy with azacitidine and venetoclax, along with intrathecal methotrexate for CNS prophylaxis. Six days following therapy completion, he presented to the emergency room with sudden-onset, left-sided chest pain. He was hemodynamically stable, and serial troponins were negative. Initial electrocardiogram (EKG) demonstrated mild ischemic changes but did not warrant acute cardiac catheterization, per cardiology review.

**Figure 1:**
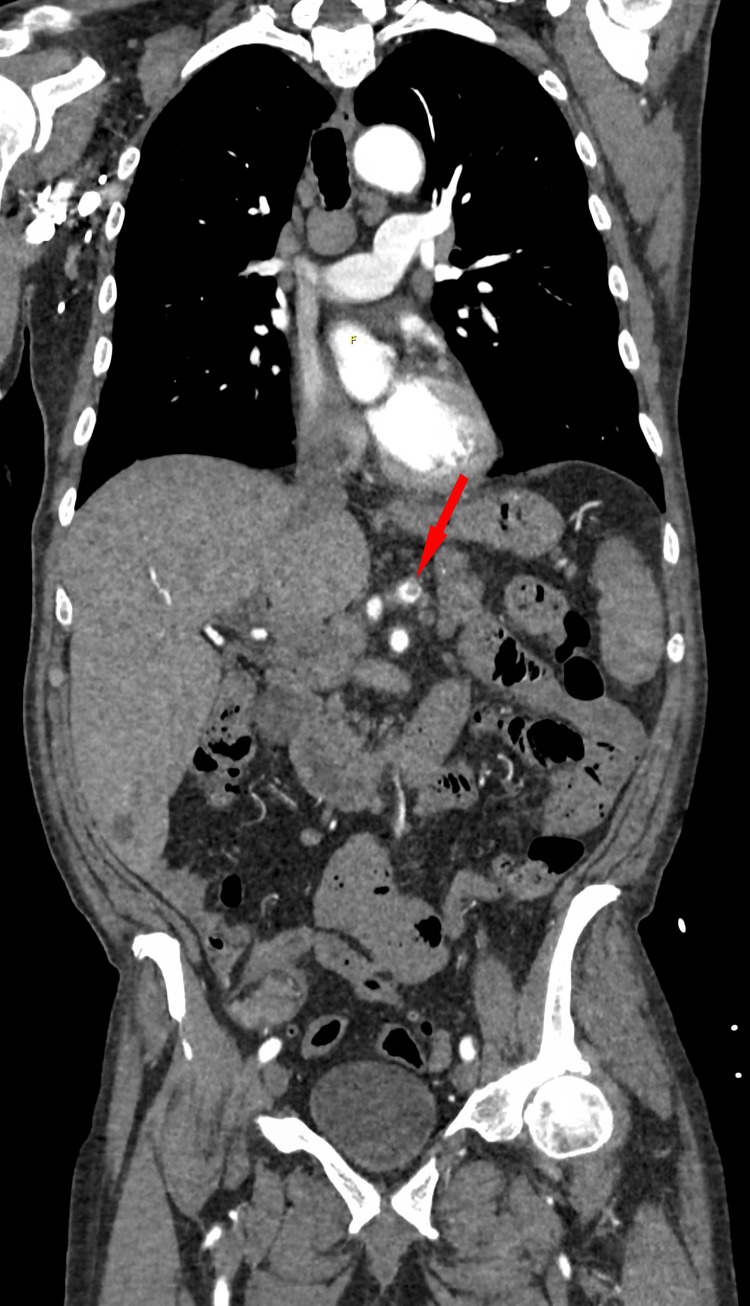
CT angiogram of the chest, abdomen, and pelvis demonstrating celiac artery occlusion due to thrombus (red arrow), and acute splenic infarct. CT, computed tomography

Laboratory studies were notable for pancytopenia: leukopenia (WBC 1.0 × 10^9^/L), anemia (Hgb 7.2 g/dL), thrombocytopenia (platelets 54 × 10^9^/L), and a markedly elevated D-dimer (35 mg/L FEU). Given a prior history of descending thoracic aortic dissection and new chest pain, a computed tomography angiogram (CTA) of the chest, abdomen, and pelvis was obtained, revealing an acute celiac artery thrombus and occlusion of the splenic artery, with an associated acute splenic infarct (Figure 1).

Despite the patient’s significant thrombocytopenia, the arterial thrombosis represented an immediate risk for bowel ischemia and further organ infarction - conditions associated with high morbidity and mortality if left untreated. After review by vascular surgery, the recommendation was for urgent anticoagulation as the most effective means to prevent thrombus propagation. The decision weighed the acute threat posed by the vascular occlusion against the elevated bleeding risk in a cytopenic patient, with the understanding that platelet counts would be closely monitored and transfusions administered as needed to reduce hemorrhagic complications.

**Figure 2:**
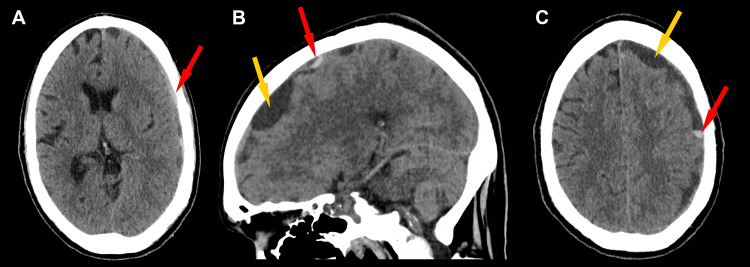
(A) Preoperative non-contrast CT of the head showing a left subdural hematoma with acute components (red arrows). (B and C) Non-contrast CT of the head, sagittal and axial views respectively, showing acute-on-chronic subdural hematoma (yellow arrows), with a notably loculated appearance due to chronic membrane formation. CT, computed tomography

Given the acute celiac and splenic artery thromboses, therapeutic heparin infusion was initiated, in line with institutional venous thromboembolism (VTE) management protocols. On admission day 3, the patient subsequently developed acute encephalopathy and disorientation. Stat non-contrast CT of the head demonstrated a large acute-on-subacute left SDH with midline shift and mass effect (Figures 2A-2C). Coagulation labs were notable for platelets 33 × 10^9^/L, PT 28.2 sec, INR 2.7, and aPTT 39.6 sec, consistent with a coagulopathic state. Intravenous protamine sulfate was given for the reversal of heparin. One unit of platelets was transfused following consultation with hematology/oncology. The patient underwent emergent left-sided craniotomy and hematoma evacuation and received transfusion products intraoperatively.

**Figure 3:**
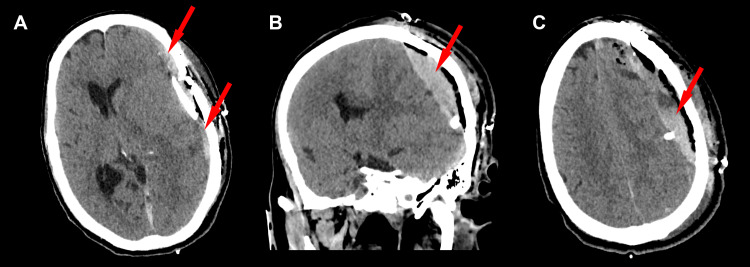
(A) Post-craniotomy day 1 non-contrast CT of the head showing new brain compression and left-to-right midline shift due to acute re-bleeding into the subdural space (red arrows). (B) Coronal view demonstrating a hyperacute left subdural hematoma re-accumulated below the craniotomy site, with diffuse interstitial cerebral edema causing uncal herniation (red arrow). (C) Acute subdural hematoma with associated pneumocephalus (red arrow). CT, computed tomography

One day later, he experienced progressive neurologic deterioration, with loss of response to voice and pain, inability to follow commands, and loss of movement in all extremities. Repeat CT head imaging at that time revealed re-accumulation of the SDH, with worsening cerebral edema and a 1.5 cm left-to-right midline shift - a magnitude reflecting substantial mass effect and brain displacement, warranting surgical intervention (Figures 3A-3C).

He was subsequently taken back to the operating room for decompressive left hemicraniectomy and repeat SDH evacuation. The patient later developed tumor lysis syndrome, leading to multi-organ failure with lactic acidosis, acute liver failure, DIC, and acute kidney injury requiring continuous renal replacement therapy. He was transferred to a tertiary care center for further management. He developed multifocal subacute cerebral infarcts, and following a multidisciplinary family discussion, life-sustaining treatments were withdrawn. The patient expired shortly thereafter.

**Figure 4:**
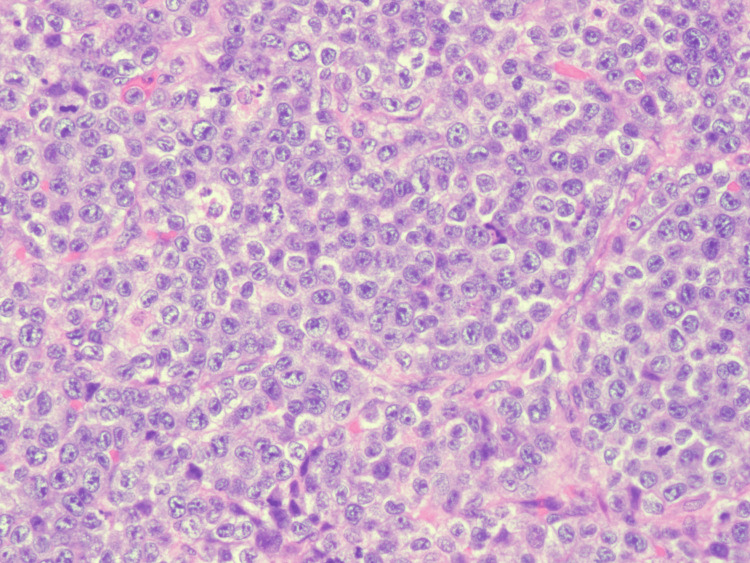
Hematoxylin and eosin stain of subdural membrane tissue demonstrating dense infiltration of immature myeloid cells.

**Figure 5:**
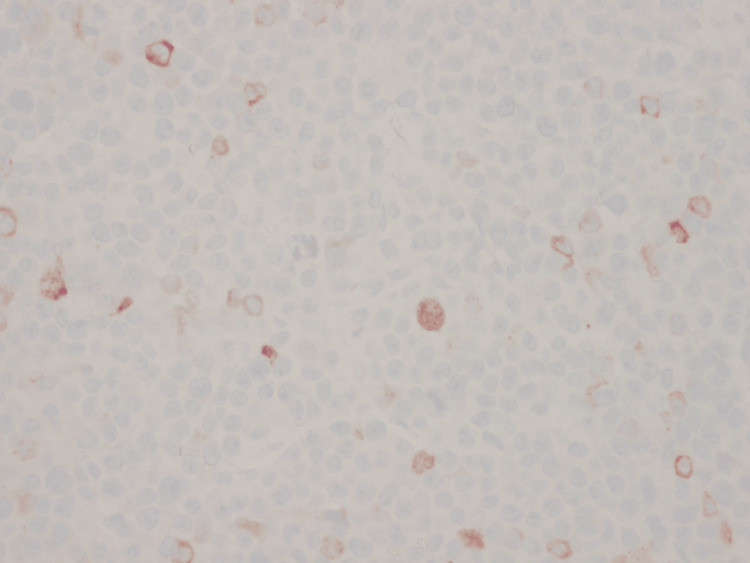
Immunohistochemical staining positive for myeloperoxidase, supporting the diagnosis of myeloid lineage infiltration.

**Figure 6:**
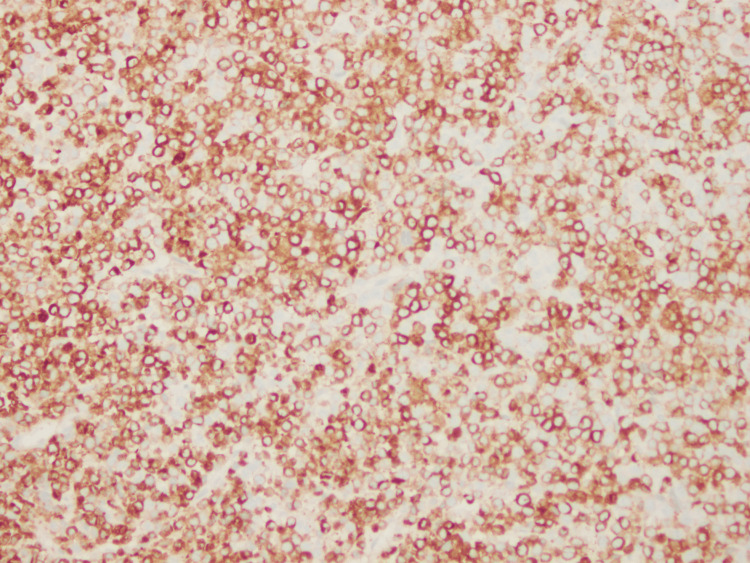
Immunohistochemistry for CD163, highlighting monocytic differentiation of infiltrating leukemic blasts. CD163 is associated with increased tissue invasiveness and extramedullary dissemination.

**Figure 7:**
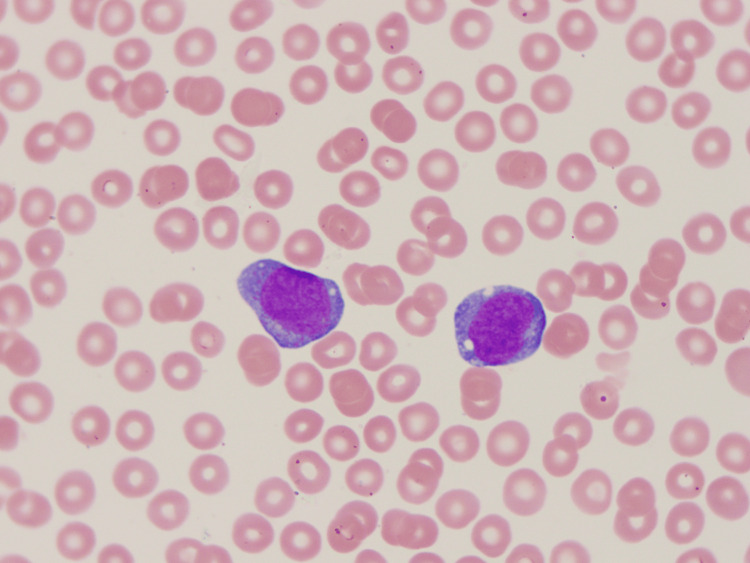
Peripheral blood smear showing circulating monocytes with abnormal nuclear morphology.

Final histopathology of the subdural membrane tissue revealed sheets of immature myeloid cells (Figure 4), with strong immunoreactivity for myeloperoxidase (Figure 5) and CD163 (Figure 6), consistent with AML with monocytic differentiation. Peripheral blood smear demonstrated monocytosis (Figure 7).

## Discussion

Chloroma, or myeloid sarcoma, is a rare extramedullary tumor composed of immature myeloid cells and may precede, accompany, or follow a systemic leukemia diagnosis. Although commonly found in soft tissues, lymph nodes, bone, and skin, CNS involvement is uncommon and often underrecognized due to its nonspecific clinical presentation and radiologic features [16]. When present in the CNS, chloromas may manifest as dural-based masses, epidural or leptomeningeal infiltration, or parenchymal lesions [3]. These can lead to neurologic symptoms, including headache, confusion, cranial neuropathies, and seizures. In rare instances, leukemic infiltration of the dura or leptomeninges may result in hemorrhagic complications, such as SDH, especially in patients who are coagulopathic.

In this case, the patient presented with a catastrophic manifestation of CNS chloroma, characterized as acute-on-subacute SDH following hypomethylating agent-based induction therapy with azacitidine and venetoclax. Several interrelated pathophysiologic mechanisms likely contributed to this outcome. First, the patient had profound thrombocytopenia and coagulopathy following induction chemotherapy, which are known adverse effects of hypomethylating agents and BCL-2 inhibitors. These cytopenias significantly increase the risk of both spontaneous and provoked hemorrhagic events, particularly in the CNS. Additionally, the patient was initiated on therapeutic anticoagulation for acute arterial thromboembolism, further compounding his bleeding risk. Finally, histopathology of the subdural membrane tissue revealed infiltration by immature myeloid cells, confirming that the SDH originated, in part, from leukemic infiltrations of the meninges, a rare mechanism of CNS bleeding in AML. The novelty of this phenomenon has further salience due to the patient’s NGS revealing a *TP53* mutation, a feature of high-risk AML associated with adverse prognosis and treatment resistance [17]. CNS involvement in AML is rare, and reported risk factors include younger age, hyperleukocytosis, elevated lactate dehydrogenase (LDH), and monocytic or myelomonocytic subtypes [18]. However, less is known about the influence of underlying cytogenetic or molecular abnormalities on the risk of CNS chloroma. To our knowledge, there are no prior reports directly linking cytogenetic abnormalities or high-risk molecular mutations, such as *TP53*, with the development of CNS chloroma. The biological mechanisms underlying such extramedullary dissemination remain incompletely understood, and this association in our patient should be interpreted with caution. This potential association warrants further investigation to elucidate the molecular mechanisms driving site-specific leukemic infiltration and to explore targeted therapeutic approaches.

Another aspect to consider is the patient having completed intrathecal methotrexate prophylaxis, which is used in AML patients at high risk for CNS involvement, such as those with monocytic or myelomonocytic subtypes [18-20]. However, the presence of CNS involvement in this case, despite prophylaxis, raises questions about the adequacy of CNS-directed therapy in preventing leukemic infiltration of the meninges. The optimal prophylactic regimen remains unclear, and some studies suggest that prophylaxis tends to delay CNS involvement rather than prevent it [21,22]. It is also unclear whether the chloroma developed de novo following therapy or represented persistent disease not recognized on initial presentation. Of note, histopathology of the subdural membrane tissue demonstrated immunoreactivity to CD163, which is associated with increased tissue invasiveness and extramedullary dissemination [23,24].

Few reports exist of chloromas presenting as SDH, but in all reported cases, delays in diagnosis were common. Patients often present with acute neurologic symptoms and undergo neuroimaging that reveals subdural collections, frequently presumed to be traumatic or coagulopathic in origin [5]. The diagnosis of CNS chloroma is rarely made preoperatively and typically requires histopathologic confirmation. This diagnostic delay may prevent early initiation of targeted chemotherapy or radiotherapy, leading to worse outcomes.

The decision to resume anticoagulation in patients with AML who develop acute thrombotic events must be carefully weighed against bleeding risk, particularly in the setting of AML-acquired coagulopathy. In this case, the dural-based chloroma likely eroded into surrounding vessels or caused venous congestion, resulting in slow hemorrhage, which matured into a subacute-chronic SDH. The patient’s acquired coagulopathy from AML, chemotherapy-induced marrow suppression, and initiation of therapeutic anticoagulation for recent thromboses likely acted synergistically to trigger catastrophic bleeding into the already-developed and previously asymptomatic SDH.

In addition to hemorrhage, the patient experienced a cascade of complications, including tumor lysis syndrome, acute liver failure, and ultimately cerebral infarction and death. These events reflect the fragile physiologic state of patients undergoing AML induction therapy and underscore the importance of aggressive supportive care, including early recognition of metabolic derangements and multi-organ dysfunction. Despite emergent neurosurgical intervention, the recurrence of SDH and progression to cerebral edema and midline shift illustrate the highly morbid nature of this condition, especially when surgical and medical therapies fail to control the underlying leukemic process.

This case advances a growing recognition that CNS chloromas can masquerade as hemorrhagic complications, especially SDH, in patients with AML. Surgical management via craniotomy and hematoma evacuation is the most appropriate treatment for patients with brain compression and decline in neurological status. However, this may be complicated by concomitant coagulopathy associated with AML, especially following induction chemotherapy. Multidisciplinary vigilance remains essential, and surgical biopsy and histology remain critical to definitive diagnosis. Immunophenotyping for monocytic markers, such as CD68, CD163, and MPO, can distinguish CNS chloroma from other neoplastic or inflammatory processes [25].

## Conclusions

We report a rare and fatal case of SDH caused by CNS chloroma in an AML patient following azacitidine/venetoclax induction and intrathecal methotrexate. In AML patients presenting with neurologic symptoms, especially those on therapeutic anticoagulation or with underlying coagulopathy, CNS chloroma should be considered in the differential diagnosis. Early multidisciplinary intervention and histologic diagnosis remain essential in guiding treatment and prognostication. While SDHs are often managed successfully with surgical evacuation, those resulting from leukemic infiltration in AML may augur a poorer prognosis due to concurrent coagulopathy and their association with more aggressive hematologic disease.

Given the diagnostic challenges and high risk of hemorrhagic complications, clinicians should maintain a low threshold for neuroimaging and consider prophylactic CNS evaluation in high-risk AML subtypes, especially those with monocytic differentiation or high-risk mutations. Future research is needed to better define screening protocols and optimize management strategies for preventing and promptly identifying CNS leukemic involvement in these vulnerable patients.
